# miR-92a-3p and miR-320a are Upregulated in Plasma Neuron-Derived Extracellular Vesicles of Patients with Frontotemporal Dementia

**DOI:** 10.1007/s12035-024-04386-z

**Published:** 2024-08-14

**Authors:** Valeria Manzini, Pamela Cappelletti, Nicola S. Orefice, Ilaria Brentari, Michael J. Rigby, Maria Lo Giudice, Marco Feligioni, Roberto Rivabene, Alessio Crestini, Francesco Manfredi, Giuseppina Talarico, Giuseppe Bruno, Massimo Corbo, Luigi Puglielli, Michela A. Denti, Paola Piscopo

**Affiliations:** 1 Department of Neuroscience, Istituto Superiore Di Sanità, Viale Regina Elena, 299, 00161 Rome, Italy; 2 Department of Biology and Biotechnology Charles Darwin, University of Rome “Sapienza”, Rome, Italy; 3 Department of Neurorehabilitation Sciences, Casa Cura Igea, Milan, Italy; 4 Department of Medicine, University of Wisconsin-Madison, Madison, WI 53705 USA; 5 Waisman Center, University of Wisconsin-Madison, Madison, WI 53705 USA; 6 Department of Cellular, Computational and Integrative Biology, University of Trento, Trento, Italy; 7 Neuroscience Training Program, University of Wisconsin-Madison, Madison, WI 53705 USA; 8 Need Institute, Foundation for Cure and Rehabilitation of Neurological Diseases, Milan, Italy; 9 Fondazione European Brain Research Institute (EBRI) Rita Levi-Montalcini, Rome, Italy; 10 National Center for Global Health, Istituto Superiore Di Sanità, Rome, Italy; 11 Department of Human Neuroscience, University of Rome “Sapienza”, Rome, Italy; 12 Geriatric Research Education Clinical Center, Veterans Affairs Medical Center, Madison, WI 53705 USA; 13 Present Address: Feinberg School of Medicine, Department of Pharmacology, Northwestern University, Chicago, IL 60611 USA; 14 Present Address: Department of Neurology, Mayo Clinic, Rochester, MN USA

**Keywords:** MicroRNA, Extracellular vesicles, Frontotemporal dementia, Alzheimer’s disease, Human iPSCs

## Abstract

Despite the efforts to identify fluid biomarkers to improve diagnosis of Frontotemporal dementia (FTD), only a few candidates have been described in recent years. In a previous study, we identified three circulating miRNAs (miR-92a-3p, miR-320a and miR-320b) differentially expressed in FTD patients with respect to healthy controls and/or Alzheimer’s disease (AD) patients. Now, we investigated whether those changes could be due to miRNAs contained in neuron-derived extracellular vesicles (NDEVs). We also evaluated miRNAs content in total plasma EVs and in CSF samples. The analysis of plasma NDEVs carried out on 40 subjects including controls (*n* = 13), FTD (*n* = 13) and AD (*n* = 14) patients, showed that both miR-92a-3p and miR-320a levels were triplicated in the FTD group if compared with CT and AD patients. Increased levels of the same miRNAs were found also in CSF derived from FTD group compared to CTs. No differences were observed in expression levels of miR-320b among the three groups. Worthy of note, all miRNAs analysed were increased in an FTD cell model, MAPT IVS10 + 16 neurons. Our results suggest that miR-92a and miR-320a in NDEVs could be proposed as FTD biomarkers.

**Supplementary Information:**

The online version contains supplementary material available at 10.1007/s12035-024-04386-z.

## Introduction

Alzheimer’s Disease (AD) represents the most common cause of dementia in the elderly, which significantly strains the healthcare and social system. The characteristic accumulation of Amyloid-β (Aβ) peptides in amyloid plaques and hyperphosphorylated Tau in neurofibrillary tangles lead to cortical and hippocampal atrophy, neurodegeneration, and activation of inflammatory pathways. Patients with AD predominantly show episodic memory impairments, while semantic memory deficits are observed to a minor degree [1].

Frontotemporal Dementia (FTD) is a heterogeneous condition characterised by atrophy in the frontal and temporal lobes of the brain [2, 3]. Clinically, patients show changes in behaviour and personality (behavioural variant FTD, bvFTD), or language impairment (primary progressive aphasia, PPA and semantic variant, svFTD).

FTD is the second most common cause of dementia and belongs to a wider group of clinical conditions called frontotemporal lobar degeneration. FTD pathological hallmarks are the hyperphosphorylated Tau and DNA binding protein 43kD (TDP-43) accumulation in the brain’s intracellular and extracellular space [4]. FTD is often misdiagnosed as a psychiatric disorder or as AD. For this reason, its real prevalence is probably underestimated, so 10–30% of FTD patients are wrongly diagnosed [5].

Thus, searching for molecular biomarkers that are easy to detect in the preclinical and clinical phases and useful for differentiating dementia etiologies represents one of the most significant challenges in research [6].

In the last decade, the scientific community highlighted the potential of small regulatory non-coding RNA molecules to be useful biomarkers for pathology diagnoses due to their high stability and ease of detection [7]. Among these, microRNAs (miRNAs) were particularly promising in neurological disorders. They act as key regulators of different biological functions, including synaptic plasticity and neurogenesis [8]. Importantly, exosomal miRNAs can cross the blood–brain barrier and be released in the cerebrospinal fluid (CSF) and blood [9]. Several miRNAs are implicated in AD pathogenesis, in particular in the interference with amyloid synthesis, aggregation, and removal, Tau phosphorylation and clearance, microglia, and astrocyte function [10, 11]. Interestingly, some studies have demonstrated that miRNA expression patterns are altered not only in the brains of patients with dementia but also in their blood samples [8, 12, 13].

Growing evidence highlighted the potential of small extracellular vesicles (EVs) as biomarkers for a variety of pathological conditions, including neurodegenerative diseases. EVs are membrane-derived vesicles characterized by a lipid bilayer membrane [14, 15] released by all cellular types including neurons [16], astrocytes [17], oligodendrocytes and microglia [18]. They are released into the extracellular milieu mediating intercellular communications [19]. They play roles in cell–cell communication including neuron-glia crosstalk, tissue development, and maintenance, immune response, apoptosis, cellular homeostasis, inflammation, and synaptic plasticity [20–25]. EVs are classified according to their size, membrane protein markers, and origins as follows: exosomes, 50–150 nm in diameter, originate from endosomal pathways and are mainly characterized by membrane CD63, CD9, and CD81; microvesicles, 150–1000 nm, which are generated by exocytosis processes, contains phosphorylation pattern typical of plasmatic membrane and express membrane receptors on their surface; apoptotic bodies, large up to 5 μm, which are released as blebs from cells undergoing programmed death and usually contain random cargoes including DNA fragments, non-coding RNAs and organelles [22, 26, 27]. EVs are found in most body fluids, transporting specific cargoes to parental cells like proteins, lipids, and nucleic acids. Focusing on the EVs fraction, miRNAs account for more than 50% of all EVs RNA [9].

Neuron-derived EVs (NDEVs) can offer a temporal-spatial picture of pathological brain alterations [28, 29]. miRNAs embedded in EVs are considered better diagnostic biomarkers than free miRNAs because their expression levels are protected from degradation by nucleases widely present in circulating fluids [30]. Several blood EV miRNAs were associated with AD [31, 32], some of which were also studied in EVs from the post-mortem AD frontal cortex [33].

In a recent study [34], we adopted an innovative approach, the microRNA-Capture Affinity Technology (miR-CATCH), to identify miRNAs targeting the *MAPT* (Microtubule-associated Protein Tau) transcript coding for Tau [35, 36]. It highlights the miR-92a-3p, miR-320a and miR-320b as possible plasma biomarkers for FTD and AD diagnosis. Particularly, the downregulation of miR-92-3p and the upregulation of miR-320b in patients with FTD or AD compared with neurologically unimpaired controls were observed. In contrast, miR-320a resulted higher in subjects with FTD than in subjects with AD, without any significant difference among controls [34]. Then, we asked whether the changes observed in plasma reflect the pathological variations occurring in the central nervous system. Thus, we evaluated miRNA contents in NDEVs and compared their levels with those of plasma small Total Extracellular Vesicles (TEVs). Moreover, the same miRNAs were quantified in CSF samples to compare their expression with those measured in NDEVs. Subsequently, to deepen the role of these three miRNAs in FTD, we extended our analysis to a human cell model derived from patients with familial FTD (FTDP-17) caused by the MAPT 10 + 16 splice-site mutation.

## Materials and Methods

### Recruitment of Patients and Healthy Controls

The studied population was enrolled by the Memory Clinic of Sapienza University (Rome, Italy), following the approval of ethical committees after all the subjects signed the informed consent. FTD diagnosis was performed following currently approved criteria [37, 38], and AD diagnosis following DSM-IV and NINCDS-ADRDA criteria [39]. Clinical and family history, physical exam, neurological examination, neuropsychological tests including Mini-Mental State Examination (MMSE), brain imaging, and laboratory tests were assessed for each patient. Healthy controls (CT) were enrolled among patients’ partners or caregivers.

### Plasma and CSF Collection

The collection of plasma samples followed previously validated procedures [40, 41]. After collecting whole blood in EDTA-containing tubes, samples were centrifuged at 1600 × *g*, 4 °C for 15 min. The upper phase, represented by plasma, was aliquoted in 250 µL and stored at − 80 °C. According to current guidelines, cerebrospinal fluid (CSF) samples were collected by lumbar puncture. CSF samples were then centrifuged at 1600 × *g*, 4 °C for 15 min, aliquoted in 250 µL, and stored at − 80 °C.

### Neuronal Differentiation of hiPSCs

Wild Type human-induced Pluripotent Stem Cells (WT hiPSCs, European Bank of Induced Pluripotent Stem Cells; depositor Sigma-Aldrich SIGi001-A-1) and relative isogenic mutated MAPT IVS10 + 16 biallelic hiPSCs (European Bank of Induced Pluripotent Stem Cells; depositor Sigma-Aldrich SIGi001-A-12) were maintained in self-renewal TeSR-E8 medium (StemCell Technologies, 05990) on Geltrex (Thermo Fisher, A1413201). The medium was replaced every other day and confluent cells were treated with EDTA (Ethylenediaminetetraacetic acid, Gibco). PSC Neural Induction Medium (Thermo Fisher, A1647801) was used to differentiate hiPSCs into neural progenitor cells (NPCs), according to the manufacturer’s instructions. Differentiation of WT and MAPT 10 + 16 p4-p7 NPCs into a mixed population of neurons was performed by seeding 30,000 cells/cm^2^ on laminin (neurons day 0) in a maturation medium composed of complete Neurobasal medium (Gibco, 21,103,049), 10 ng/ml BDNF (Brain-derived neurotrophic factor, PeProtec, 450–02), 10 ng/ml GDNF (Glial cell line-derived neurotrophic factor, Peprotec, IVS450-10) and 200 nM Ascorbic Acid (PeProtec, 5,088,177). Change of medium took place twice a week until the desired age was reached. All the cells were incubated in a humified incubator at 37 °C with 5% CO_2_.

### Extracellular Vesicle Purification and Characterization

EVs were isolated from plasma samples as previously described [28, 42]. Briefly, 500 µL of plasma was added to 500 µL of Phosphate Buffered Saline solution (PBS), supplemented with 3 times concentrated protease and phosphatase inhibitors cocktail (ThermoScientific) and centrifuged at 4000 × *g* for 20 min at 4 °C. 250 µL of ExoQuick (System Biosciences) was added to supernatants and samples were incubated for 1 h at 4 °C and then spun down at 1500 × *g* for 20 min at 4 °C. Pellets were resuspended in 500 µL of Ultra-Pure Water (Lonza Bioscience Solution) with protease and phosphatase inhibitors (300 ×) and incubated for 2 h at room temperature (RT). 100 µL of samples (TEVs) were collected and divided: 50 µL of sample was added with RIPA Buffer (Thermo Scientific) supplemented with 3 times concentrated phosphatase and protease inhibitors for following Western Blot analysis, and 50 µL of sample was added with RNA Later™ (Qiagen) for following miRNAs expression analysis. NDEVs were immunoprecipitated with 4 µg of mouse anti-human CD171 (L1 cell adhesion molecule [L1CAM] biotinylated antibody from eBiosciences) in 45 µL of 3% BSA (Bovine Serum Albumine) in PBS and incubated for 1 h on a rotating wheel. Samples were centrifuged at 200 × *g* for 10 min at 4 °C, and supernatants, representing TEVs depleted of NDEVs (T-N EVs), were subdivided as described above for TEVs. Pellets (containing NDEVs) were resuspended in 160 µL of 0.1 M Glycine, pH 2.5–3, centrifuged at 4500 × *g* for 5 min at 4 °C. Supernatants were added of 13.5 µL of 1 M Tris–HCl, pH 8.0 and 22.5 µL of 3% BSA in PBS, and 42 µL of samples were added of RIPA Buffer for Western Blot analysis, while 146 µL of NDEVs were added of 146 µL RNA Later™ for miRNAs analysis.

EVs particle size and concentration were evaluated by Nanoparticle Tracking Analysis (NTA) using a Nanosight NS300 instrument (Malvern Panalytical, Malvern, UK) equipped with a 488-nm laser and a syringe pump system. Thawed NDEVs and TEVs fractions from the plasma of two healthy controls were diluted 1:600 in filtered PBS before NTA analysis, and five videos were taken for each EVs preparation. The buffer used for EV dilution was checked for purity and used as a baseline. Captured video recordings were analysed using the NTA 3 software version to obtain the concentration (particles/mL) and the size distribution curves.

### Plasma EVs Protein Quantification and Western Blot Analyses

EV fractions, supplemented with RIPA Buffer, were subjected to 2 freeze–thaw cycles, sonication, and determination of protein concentration by using Coomassie Protein Assay (Thermo Scientific). 100 µg of EVs and 4 µg of mouse brain cortex (positive control) lysates were diluted in Laemmli Loading Buffer (WVR Life Science) and loaded for western blot analysis performed by incubating polyvinylidene difluoride (PVDF) membranes (GE Healthcare) for 1 h at RT or overnight at 4 °C in blocking solution containing 4% of dried milk (Serva Electrophoresis GmBH) or BSA (PanReac AppliChem) in TBS (Tris-Buffered Saline, Corning) to which 0.1% Tween-20 was added. After incubation with anti-Neuron-Specific Enolase (NSE—1:400, Biorbyt), anti-CD9 (1:200, Elabsciences), anti-L1 Cell Adhesion Molecule (L1CAM—1:500, Antibodies.com) and anti-Proteolipid Protein 1 (PLP1 – 1:1000, Clinisciences) for 90 min at RT or overnight at 4 °C, membranes were extensively washed in TBS and 0.1% Tween-20 and incubated for 1 h at RT with anti-rabbit peroxidase-conjugated immunoglobulins (Jackson ImmunoResearch). The immunoreactivity signals were detected by Super Signal^TM^WestFemto Maximum Sensitivity Substrate (ThermoScientific), images were acquired using Azure C300 Gel Imaging System (Bio-System), and densitometric analysis was performed using ImageJ software (MeidaCybernetics).

### RNA Extraction and Expression Analysis of miRNAs

The miRNeasy Serum/Plasma Kit (Qiagen) was used for miRNA extraction from plasma-derived EVs, CSF, and culture medium of neurons derived from hiPSC, assuring the enrichment of small molecules like miRNAs [43]. According to the kit protocol, 5 volumes of Qyazol Lysis Reagent were added to the samples and incubated for 5 min at RT; chloroform at an equal volume to the starting sample was added and incubated for 2–3 min at RT. After centrifuging for 15 min, 12,000 × *g*, at 4 °C, the upper phase was collected and mixed with 1.5 volumes of 100% ethanol. Samples were filtered with the supplied RNeasy MinElute spin columns at 8000 × *g*, for 15 s. The columns were washed at 8000 × *g*, for 15 s with 700 μl of RWT Buffer and 500 μl of RPE Buffer, and at 8000 × *g*, for 2 min, with 500 μl of 80% Ethanol. RNA was eluted with 14 μl of RNase-free water.

Regarding the cultured neurons, total RNA was isolated by using TRIzol Reagent (Life Technologies, 15,596,026) according to manufacturer instructions. Briefly, cells were washed with 1X PBS and lysate with 500 µL of TRIzol directly in the plate. Then, 100-μL chloroform was added to each lysate, tubes were vortexed vigorously and then centrifuged at 12,000 × *g* at 4 °C for 15 min. The aqueous phase containing RNAs was collected in new tubes. To precipitate RNA, 250-μL cold propan-2-ol and 1-µL RNAse-free glycogen were added to the samples and incubated in ice for up to 1 h. A centrifugation step was performed at 12,000 × *g* at 4 °C for 15 min during which the pellet became visible. After the removal of the supernatant, the pellet was rinsed with 75% ethanol and centrifuged at 8200 × *g,* for 10 min at 4 °C. After discarding the supernatant and having let the ethanol evaporate, RNA was resuspended in RNase-free water.

Each RNA concentration was measured via a UV–Vis spectrophotometer (NanoDrop ND-1000, Thermo Fisher; Supplementary Table 1).

### Real-Time PCR

For plasma and CSF total RNA, cDNA was obtained using ID3EAL cDNA synthesis reagents (MiRXES, Singapore) with modified stem-loop reverse transcription primer pool for miR-92-3p, miR-320a, and miR-320b and 3 exogenous spike-in controls (MiRXES, Singapore). Total RNA was mixed with ID3EAL miRNA reverse transcription buffer, ID3EAL reverse transcriptase, and reverse transcription primer pool in a total reaction volume of 10 μL. The reaction mixture was incubated at 42 °C for 30 min, followed by 95 °C for 5 min to inactivate the reverse transcriptase. According to the manufacturer’s protocol, real-time quantitative PCR (RT-qPCR) was performed using ID3EAL miRNA qPCR reagents (MiRXES, Singapore). Each cDNA sample was diluted ten times with nuclease-free water. PCR amplification was performed in a total reaction volume of 10 µL containing 5-µL diluted cDNA, 1 X ID3EAL miRNA qPCR master mix, 1 X ID3EAL miRNA qPCR primers (MiRXES, Singapore), topped up with nuclease-free water. qPCR amplification and detection were performed on ABI PRISM 7500 (Thermo Fisher Scientific) with the following cycling conditions: 95 °C for 10 min, 40 °C for 5 min, followed by 40 cycles of 95 °C for 10 s, and 60 °C for 30 c. As a reference, to normalize the expression of the analyzed miRNAs, we selected miRNAs, checking their expression levels and stability in our samples by using the NormFinder [44, 45]: miR-16 was the most stable among the selected miRNAs.For cells and culture medium, the analysis of microRNAs was performed employing the TaqMan® MicroRNA Assays protocol (Thermo Fisher), according to the manufacturer’s instructions. In particular, 10-ng total RNA was retrotranscribed using microRNA-specific retrotranscription primers (Thermo Fisher) for cDNA synthesis. The mix contained, other than the input RNA, 5 μL of stem-loop retrotranscription primer, 1.5 μL of 10X RT buffer, 0.15 μL of 100 mM dNTPs, 1 μL of 50 U/μL MultiScribe reverse transcriptase, and 4.16 μL of nuclease-free water. This reaction was incubated into T100 Thermal Cycler (Biorad) for 30 min at 16 °C, 30 min at 42 °C, and 5 min at 85 °C. Quantitative PCR was performed in 20 μL containing 1.33 μL of miRNA-specific cDNA, 10 μL of FastStartTaqMan Probe Master (Roche, 04673417001), 7.67 μL of nuclease-free water, and 1 μL of TaqMan MicroRNA Assay (Thermo Fisher, Supplementary Table 2). Reactions were incubated at 95 °C for 10 min, followed by 40 cycles of incubation at 95 °C for 15 s and at 60 °C for 1 min. Reactions were performed in Quantstudio5 384 real-time detection system (Thermo Fisher) and results were evaluated with related software (Thermo Fisher). To normalize the expression of the analyzed miRNAs, we selected miRNAs, checking their expression levels and stability in our samples by using the NormFinder, RNU48 was chosen as an endogenous control to normalize microRNAs expression in cells, while cel-miR-39 was used for medium-derived miRNAs.

In each assay, all measurements were done in technical triplicates and negative controls were included. Data of RT-qPCR were expressed as 2e(-ΔCt).

### Amyloid and Tau Detection in CSF

Amyloyd-beta (Aβ) 40 and 42 isoforms, total (tTau) and phosphorylated at residue 181 (pTau181) Tau were detected in CSF samples by Lumipulse G600II (Fujirebio). Samples were treated and analysed according to the manufacturer’s protocols supplied with the kits Lumipulse G Aβ-40 (231,524), Aβ-42 (230,336), tTau (230,312) and pTau181 (230,350) for CSF: AD patients had higher tTau and pTau181 concentration values and lower Aβ42/40 ratios (Table 1).

**Table 1:** Demographic and clinical characterization of the studied population. Data were shown as mean ± standard deviation. MMSE: Mini-Mental State Examination (Score ≤ 18: severe cognitive impairment;19–25: moderate cognitive impairment; 26–30 normal cognition)

	N (M/F)	Age (Mean ± SD)	Age at onset (Mean ± SD)	MMSE (Mean ± SD)	Aβ-42/40 (Mean ± SD)	tTau (pg/ml) (Mean ± SD)	pTau181(pg/ml) (Mean ± SD)
CT	13 (7/6)	74.7 ± 8.1	‒	‒	0.123 ± 0.01	302.0 ± 27.23	29.1 ± 6.85
FTD	13 (6/7)	73.7 ± 7.3	69.6 ± 7.1	15.7 ± 5.7	0.113 ± 0.0 1	235.14 ± 51.00	30.71 ± 4.27
AD	14 (4/10)	72.8 ± 2.1	64.1 ± 6.5	18.2 ± 5.0	0.049 ± 0.01	711.8 ± 264.97	123.76 ± 56.31

### Statistical Analysis

One-way ANOVA analysis followed by post hoc Tukey significance tests were used to evaluate the NSE and CD9 expression levels in both NDEVs and TEVs (Kaleidagraph software).

The ΔCt values of miRNA levels were expressed as mean ± standard error. We applied the Student’s *T*-test to obtain the *p* values and compare differential expressions between two groups (*p* ≤ 0.05). To compare multiple groups, we used ANOVA test and Bonferroni post hoc. The Pearson test was used for the correlation analysis.

The Spearman test (by GraphPad Prism 9 software) was used to correlate the CSF protein biomarkers and miRNA levels from CSF.

## Results

### Samples’ Characteristics

For this study, we enrolled a population of 40 subjects including 13 CT, 6 females and 7 males (mean age 74.7 ± 8.1); 14 patients with AD, 10 females and 4 males (mean age 72.8 ± 2.1); 13 with FTD, 7 women and 6 men (mean age 73.7 ± 7.3). All patients were sporadic, and no mutations were found in the genes most involved in AD and FTD, such as *APP*, *PSEN1*, *PSEN2*, *MAPT*, *GRN*, and *C9ORF72*. All the characteristics of the enrolled population are summarized in Table 1.

### Characterization of Total and L1CAM Positive EVs from Plasma

**Fig. 1:**
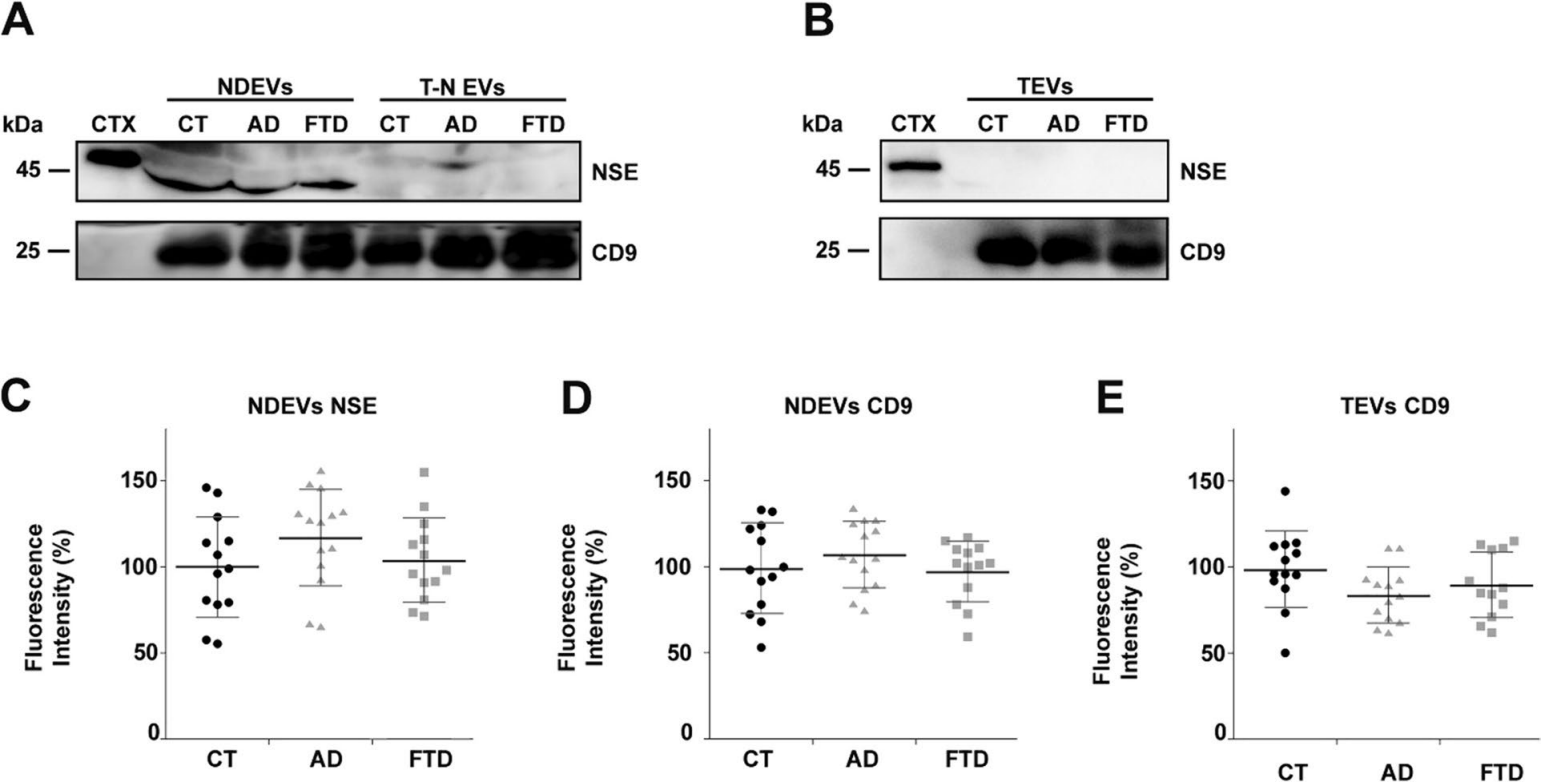
Western blot analysis of a representative NDEVs purification from plasma of CT, AD and FTD subjects**. **(**A**) An enrichment of neuronal marker (NSE) is observed in the NDEVs fraction concerning T-N EVs, (**B**) which is undetectable in the TEVs fractions. CD9 is a common exosome marker. 4 µg of mouse brain cortex (CTX, positive control) and 100 µg of EVs have been loaded in each lane. (**C**, **D**, **E**) Densitometric analysis of NSE in NDEVs (**C**), CD9 in NDEVs (**D**), and CD9 in TEVs (**E**). NSE and CD9 expression levels have been analysed using One-way ANOVA with a post hoc Tukey test. No differences are observed for the number of EVs extracted (both NDEVs and TEVs) in the CT group concerning AD and FTD ones. Values are expressed as % concerning the CT group. Each point in the frame depicts the value for a single subject, while bars represent the median value ± standard deviation. Each represents the mean of 2–3 replicates.

NDEVs were purified from the plasma of 13 CT subjects, 14 AD and 13 FTD patients. In parallel, the T-N EVs and TEVs fractions were obtained. The NDEVs fraction was indeed enriched with the NSE neuronal marker thus confirming the Central Nervous System (CNS) origin of these vesicles, which was absent in the T-N EVs fractions (Fig. 1A). In contrast, all vesicles were positive to CD9 antibody, which is specific for exosomal populations (Fig. 1A, Fig. 1B). We recently demonstrated the quality of our NDEV preparations with a characteristic morphology observed by TEM and Western Blot analysis that did not reveal the presence of any proteins derived from cell organelles [42]. Moreover, the quality of EVs preparations was further tested by using an additional neuronal marker (L1CAM) [28] and an oligodendrocyte marker (PLP1) [46, 47]: an enrichment of L1CAM was observed only in NDEVs fractions (and was undetectable in T-N EVs and TEVs fractions), while the PLP1 signal was not detected in any fractions (Supplementary Fig. 1), thus confirming that the extraction of NDEV was successfully enriched in neuronal vesicles without oligodendroglial contamination. Furthermore, the densitometric analysis showed no differences in the amount of EVs (expressed as % to the CT group) extracted between groups (Supplementary Table 3). In addition, NSE expression levels are comparable in NDEVs fractions. Similarly, CD9 expression levels are comparable in both NDEVs and TEVs fractions (Fig. 1C, Fig. 1D, Fig. 1E, Supplementary Table 3).

**Fig. 2:**
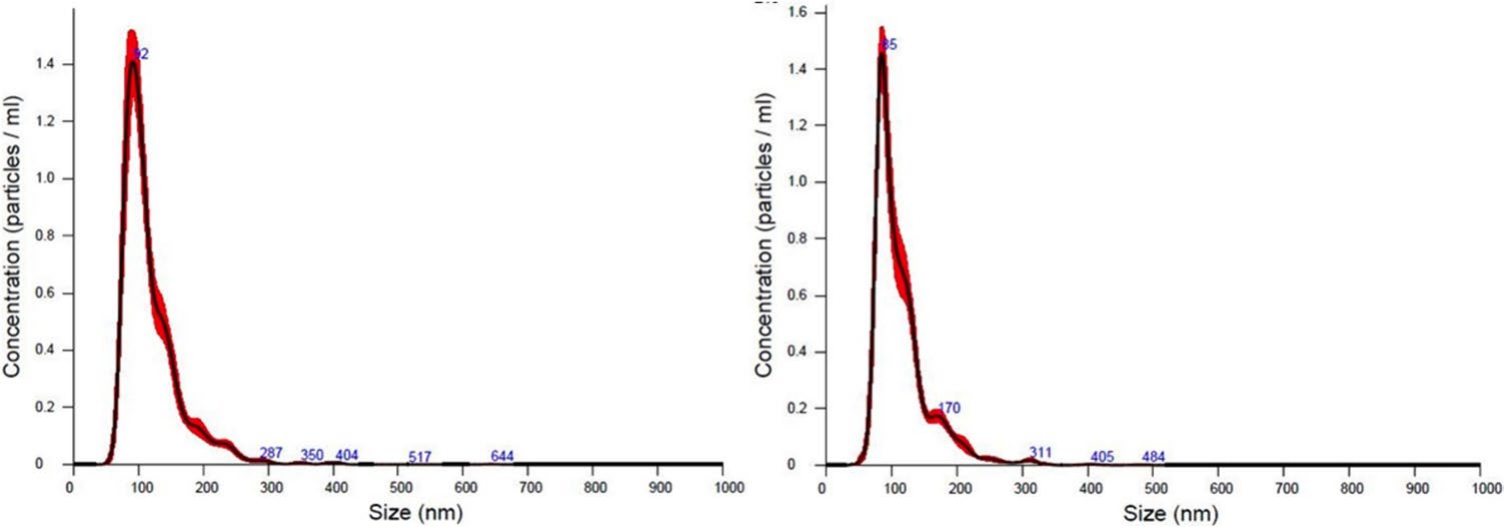
Nanoparticle tracking analysis of both size distribution and relative concentration of microvesicles. TEVs were isolated from plasma samples (1 mL) collected from two healthy volunteers. As expected for the exosomal fraction, both plots show that the majority of the EV population is distributed between 50 and 150 nm

The EVs were characterized by Nanosight 300 (Fig. 2). Considering the two analysed healthy controls, the concentration measurements of TEVs obtained from 0.5 mL of plasma were respectively 8.6 ± 0.3 × 10^11^ and 7.5 ± 0.28 × 10^11^ particles/mL. D-values showed that 10%, 50%, and 90% of the size distribution were below 78.6 ± 1.0 nm, 104.7 ± 1.2 nm, and 173.8 ± 1.9 nm to the first subject, and below 76.9 ± 0.7 nm, 102.1 ± 1.5 nm and 170.6 ± 1.5 nm respectively to the second. These data suggest that the predominant particles from the plasma samples display a typical exosomal trait.

### miR-92a-3p, miR-320a, and miR-320b Levels in Plasma TEVs and NDEVs

After EVs purification, we investigated the differential expression of miR-92a-3p, miR-320a, and miR-320b, selected from our previous study [34], among patients with AD, FTD and CT in plasma NDEVs and TEVs samples.

**Fig. 3:**
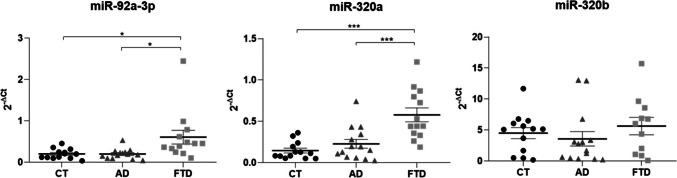
Scatter plots of the miRNA levels in plasmatic NDEVs from CT, AD and FTD groups. Relative quantification of miRNAs in FTD and AD patients compared to CTs in NDEVs. The bold bars represent the average value ± standard error. *p < 0.05; ***p ≤ 0.001

Although the miR-92a-3p and miR-320a levels were very similar in NDEVs between CT (miR-92a-3p: 0.200 ± 0.03; miR-320a: 0.145 ± 0.03) and AD (miR-92a-3p: 0.198 ± 0.03, CTvsAD, *p* = 0.957; miR-320a: 0.228 ± 0.03, CTvsAD, *p* = 0.195) groups, both of them triplicated in the subjects with FTD (miR-92a-3p: 0.605 ± 0.17; miR-320a: 0.578 ± 0.08), reaching *p*_values of 0.026 and 0.001 compared with CT group, and 0.020 and < 0.001 with respect AD group, respectively for miR-92a-3p and miR-320a (Fig. 3, Supplementary Table 4). On the contrary, no difference was observed in miR-320b expression levels among the three groups (CT: 4.503 ± 0.89, AD: 3.570 ± 1.17, FTD: 5.639 ± 1.41; CTvsAD, *p* = 0.537; CTvsFTD, *p* = 0.490; ADvsFTD, *p* = 0.266) as graphed in Fig. 3 and Supplementary Table 4. The data remained significant after Bonferroni correction for miR-320a, but not for mir-92a-3p.

**Fig. 4:**
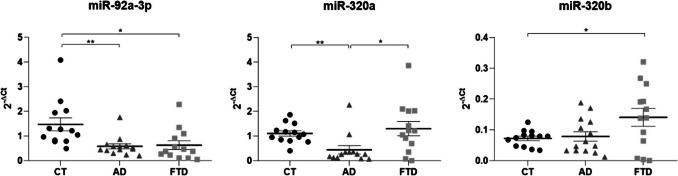
Scatter plots of the miRNA levels in plasmatic TEVs from CT, AD and FTD groups. Relative quantification of miRNAs in FTD and AD patients compared to CTs in TEVs. The bold bars represent the average value ± standard error. *p < 0.05; **p ≤ 0.01

Considering the plasma-derived TEVs (Fig. 4, Supplementary Table 5), we found similar levels of miR-92a-3p between groups of patients (AD: 0.580 ± 0.10, FTD: 0.629 ± 0.18; ADvsFTD, *p* = 0.809), while they were halved when compared to healthy controls (CT: 1.474 ± 0.27), with a statistical significance of *p* = 0.004 for AD and *p* = 0.014 for FTD. Otherwise, the miR-320a expression in CTs (1.105 ± 0.11) was similar to those in the FTD group (1.303 ± 0.29; CTvsFTD, *p* = 0.527), while it was halved in the AD group (0.441 ± 0.17; CTvsAD, *p* = 0.003; FTDvsAD, *p* = 0.016). Regarding miR-320b, patients with FTD had higher levels than controls and the AD group (CT: 0.073 ± 0.01, AD: 0.079 ± 0.01, FTD: 0.141 ± 0.03), but they reached the statistical significance only if compared to CTs with a *p*_value of 0.031 (CTvsAD, *p* = 0.719; ADvsFTD, *p* = 0.065; ADvsFTD, *p* = 0.719) as reported in Fig. 4 and Supplementary Table 5. The data remained significant after the Bonferroni correction except for mir-320b.

### miR-92a-3p, miR-320a and miR-320b Levels in CSF Samples

**Fig. 5:**
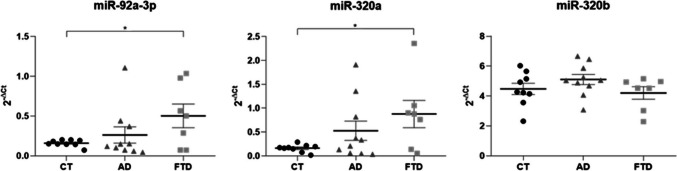
Scatter plots of the miRNA levels in CSF from CT, AD and FTD groups. Relative quantification of miRNAs in FTD and AD patients compared to CT in CSF samples. The bold bars represent the average value ± standard error. *p < 0.05

To investigate if the miR-92a-3p, miR-320a and miR-320b found in peripheral plasma reflect their expression in a fluid directly derived from the central nervous system, the expression of the same miRNAs was also detected in CSF from AD and FTD patients as well as CT subjects. Both miR-92a-3p and miR-320a showed higher levels in the FTD group (miR-92a-3p: 0.502 ± 0.15; miR-320a: 0.877 ± 0.29) with respect to CTs (miR-92a-3p: 0.160 ± 0.01; CTvsFTD, *p* = 0.021; miR-320a: 0.160 ± 0.03; CTvsFTD, *p* = 0.013), but none of them significantly differed from patients with AD (miR-92a-3p: 0.262 ± 0.10; CTvsAD, *p* = 0.365; ADvsFTD, *p* = 0.189; miR-320a: 0.527 ± 0.20; CTvsAD, *p* = 0.109; ADvsFTD, *p* = 0.320). Moreover, mir-320b showed similar levels in all the three groups analyzed, as reported in Fig. 5 and Supplementary Table 6 (CT: 4.478 ± 0.38, AD: 5.108 0.34, FTD: 4.205 ± 0.42; CTvsAD, *p* = 0.112; CTvsFTD, *p* = 0.365; ADvsFTD, *p* = 0.229). The data remained significant after the Bonferroni correction only for mir-320a.

### Correlation Between CSF Protein Biomarkers and miRNA Levels

CSF samples were analysed by Lumipulse G600II (Fujirebio) to measure the protein biomarker levels. As expected, the AD patients had higher concentrations of tTau and pTau181 and lower values of Aβ-42/40 ratio than the FTD and CT groups, as shown in Table 1. Then, we correlated the protein biomarkers with miRNA expression levels in CSF into CT, AD and FTD groups. The Spearman correlation coefficients and the relative *p*_values were reported in Supplementary Fig. 2. Interestingly, the miR-92a-3p positively correlated with the Aβ-42/40 ratio (*r* = 0.759; p = 0.015), while the miR-320a showed a positive correlation with the tTau levels in FTD patients (*r* = 0.821; *p* = 0.030).

### miR-92a-3p, miR-320a and miR-320b in Neurons Derived from Human hiPSCs with Biallelic MAPT IVS10 + 16 Mutation

Neurons derived from human hiPSCs with biallelic MAPT IVS10 + 16 splicing mutation were used as a FTD model, and results were compared to those obtained from the corresponding isogenic wild-type hiPSCs [48]. Mature neurons derived from 10 + 16 hiPSCs with 120-day-long differentiation protocol are obtained to recapitulate several FTD hallmarks: 4R/3R Tau unbalance, neurodegenerative and neurodevelopmental phenotypes [48] and impaired neuronal excitability [49, 50].

**Fig. 6:**
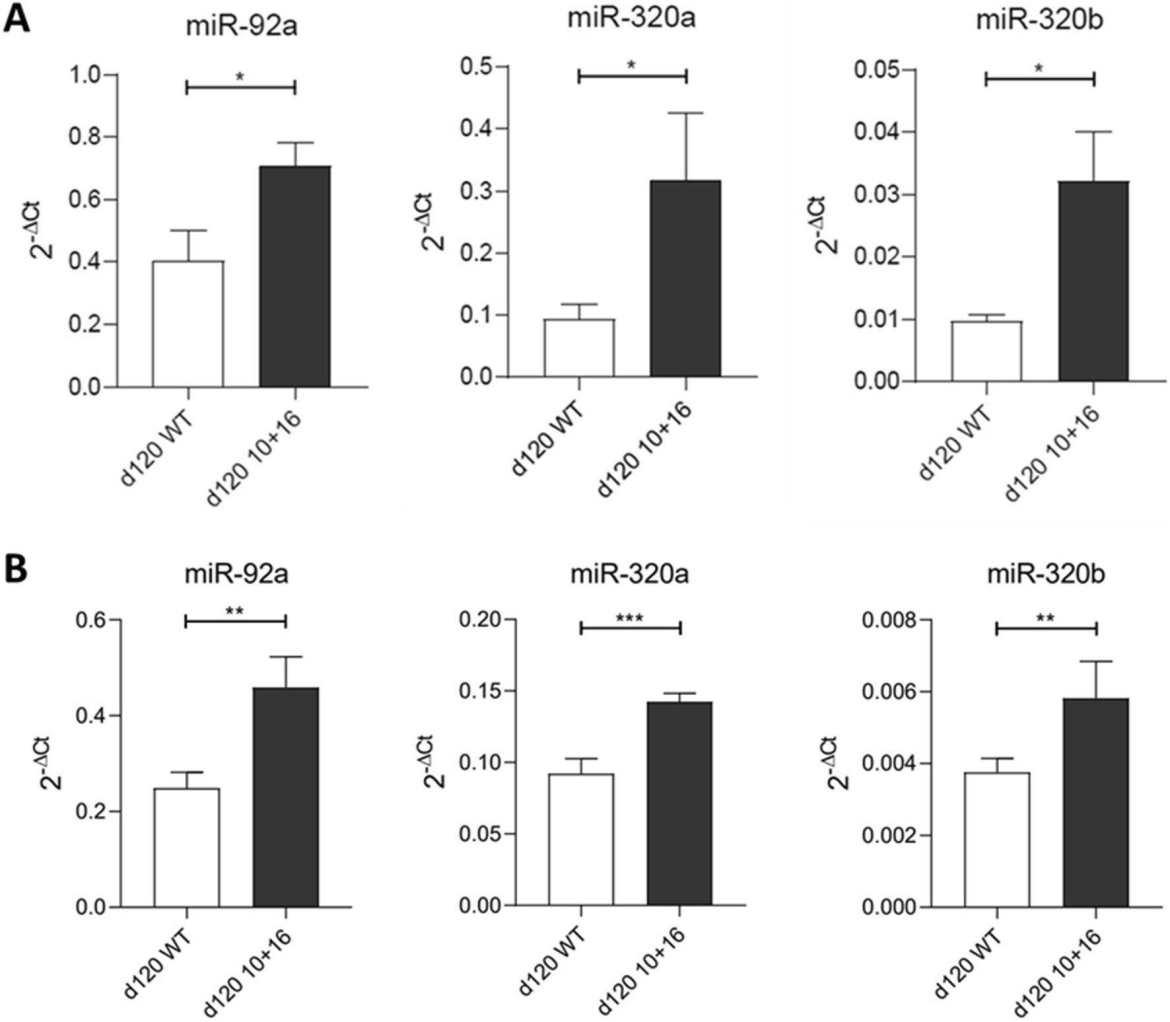
miRNA levels in FTD cellular model. Quantification of the miRNA levels (expressed as 2^-ΔCt) in (A) neurons and (B) culture medium derived from hiPSCs. White columns are referred to wild-type hiPSC, while the black ones to the MAPT IVS10 + 16 mutated hiPSCs, after 120 days from differentiation inputs (*n* = 4 for each group). *p < 0.05; **p < 0.01; ***p < 0.001

We measured the levels of the three miRNAs (normalized on snoRNA U48 expression) in these FTD neurons and observed a significant upregulation compared to healthy neurons at 120 days of differentiation. In particular, miR-320a and miR-320b levels triplicated in mutated neurons, while miR-92a-3p levels increased by approximately 1.75 times (Fig. 6A). To further confirm these results, we measured miRNAs also in the culture medium. As shown in Fig. 6B, all the three miRNAs were upregulated in MAPT IVS10 + 16 mutated than the wild-type hiPCS.

## Discussion

The importance of small non-coding RNAs as post-transcriptional regulators of pathology-related genes, including neurodegenerative diseases, is lately emerging. The intrinsic properties of miRNAs, such as their high stability and ease of detection, make them good candidates as diagnostic and prognostic biomarkers. Moreover, they are found in peripheric biofluids, such as blood or plasma, where they could reflect the physiological or pathological state. Interestingly, even more studies correlate several miRNAs with the diagnosis or progression of neurodegenerative diseases. In our previous study, we proposed three different miRNAs as diagnostic plasma-biomarkers candidates for AD and FTD: miR-92a-3p, miR-320a and miR-320b [34]. They were selected by using the miR-CATCH methodology on the* MAPT* transcript [51]. Currently, we focused our work on the analysis of the expression of the same miRNAs in plasma-derived neuronal extracellular vesicles, considering that NDEVs isolated from plasma may be used as a source of miRNAs reflecting a particular pathological condition of the nervous system.

In NDEVs, we found miR-92a-3p and miR-320a up-regulated in FTD patients, but not in AD patients; unlike what we observed in plasma samples, specifically for the miR-92a-3p, downregulated in patients. Interestingly, the induction of miR-92a-3p and miR-320a in NDEVs from patients with FTD was also significant with respect to the AD group. Contrarily, miR-320b was up-regulated in plasma, but we did not note some differences in NDEVs. To better understand whether these results were specific to neuronal extracellular vesicles, we extended the analysis to total extracellular vesicles and CSF as well. As expected, the data on TEVs were similar to those observed in plasma, leading us to speculate that the up-regulation of miR-92a-3p and miR-320a observed in NDEVs from FTD patients was brain-derived. Worthy of note, the increased levels of these miRNAs also in CSF from FTD patients confirmed our hypothesis. Furthermore, we correlated the miRNA expression in CSF with the levels of the canonical protein CSF biomarkers for AD diagnosis. Specifically, we measured the concentration of tTau and pTau181, which indicate the formation of the neurofibrillary tangles and thus the cellular death, and the ratio Aβ42/40 for the amyloid accumulation and deposition. Interestingly, we found that miR-320a positively correlates with tTau levels in FTD group suggesting the existence of indirect regulatory mechanisms between the miR-320a and the Tau expression.

To further deepen our findings, we analysed miRNA expression in hiPSCs-derived neurons. In particular, we induced the differentiation in neural progenitor cells of the wild-type hiPSCs and relative isogenic mutated MAPT IVS10 + 16 biallelic hiPSCs. All three miRNAs analysed were increased in the mutated model compared to the wild type confirming the results obtained in NDEVs for mir-320a and mir-92a-3p, while mir-320b was upregulated in hiPSCs-derived neurons, but not in NDEVs.

These results are consistent among them, confirming that the differences in miRNA levels seen in the plasma of patients with AD [34] likely do not stem from the brain. The discrepancy between the results obtained on plasma and those obtained on NDEV could be due to the very low percentage of vesicles derived from the brain in the blood. Li and colleagues have developed an algorithm to define the origin of extracellular vesicles, based on membrane markers: their results indicated that only 0.65% of the vesicles present in blood come from the brain [52]. Indeed, the miRNAs analyzed in our previous study are not only derived from the brain but also other tissues, as described on the website TissueAtlas (uni-saarland.de). Effectively, our results suggest that miRNA levels identified in plasma may have an origin other than the neuronal one demonstrating that plasma NDEVs could represent a distinct and more accessible source of CNS biomarkers. Worthy of note, it should not exclude a relation between non-neural miRNAs and neurodegenerative diseases. The glial cells play a fundamental role in neuro-pathological processes, and they can release EVs into the blood [53, 54].

In the last few years, the role of miRNAs released into EVs has aroused considerable interest. They may regulate the expression also in the EVs-receiving cells, contributing to the pathology’s spread or inhibition, depending on the type of miRNA cargoes [55]. Consequently, the biomarker potential of EVs-derived miRNAs is increasingly accepted [55]. Since miRNAs are released in EVs by cells, they may also reflect their pathological state [56]. Interestingly, as the changes that occur in the CNS can be reflected in the periphery, it has also been hypothesized that peripheral inputs may affect processes that occur in the CNS, establishing bidirectional communication [57]. Therefore, microRNAs of various origins can also influence the pathogenesis of neurodegenerative diseases and be involved in both AD and FTD.

Despite that, there are not many articles in the literature on EVs miRNAs and dementia. Yang and colleagues identified few miRNAs in serum EVs, able to discriminate AD from vascular dementia and Parkinson’s disease [58]. In another study, Wei et al. identified a subset of potential diagnostic miRNA biomarkers that correlated with some of the clinical scales used for AD diagnosis [59]. Finally, the miR-485-3p contained in salivary exosomes was associated with amyloid-β accumulation in the brain in subjects with AD [60]. In the last year, Visconte et al. identified a panel of EVs-derived miRNAs deregulated in the plasma of AD patients, including miR-92a-3p, which was up-regulated also in prodromal AD [61]. Our results are not in accordance with these reports. These discrepancies are probably due to the differences in the studied population and methodology.

Regarding FTD, only few studies investigated the role of miRNAs in EVs. One interesting study analysed the exosomal miRNAs in CSF of patients from the Genetic FTD Initiative (GENFI) with sporadic FTD, highlighting the downregulation of the miR-632 and the miR-204-5p in patients [62]. They recognised a good potential for the miR-632 in the diagnosis of the genetic and sporadic form of FTD, while the miR-204-5p appeared to have an interesting diagnostic potential only for the genetic FTD [62]. On the other hand, Pounders and colleagues examined the expression of miRNAs in NDEV among FTD and AD patients and CT subjects: their results indicated that miR-181c was downregulated in FTD patients to CT subjects, while miR-122 and miR-3591 were downregulated in AD patients with respect to FTD and CTs [63].

Interestingly, even more exosome-derived miRNA detection methods were developed by the most recent technologies, to optimize the sensitivity and minimize the costs of detection [64]. For example, Song and colleagues developed a biosensor able to build a precise profile of exosomal miRNAs with a diagnostic aim for AD and mild cognitive impairment [65].

The most interesting finding that emerges from this study is that miR-92a-3p and miR-320a could be potential biomarkers for differential diagnosis between AD and FTD patients. Their levels are three times higher in patients with FTD. Biomarkers that accurately predict the specific biochemical type of pathology in individuals with FTD are currently lacking. To date the development of CSF biomarkers based on neuropathological profiles can help to discriminate FTD from other types of dementia [66–68], even if Tau/Ab42 ratio can distinguish AD from FTD with a diagnostic accuracy of only 70% for the bvFTD forms [69]. Moreover, no biomarker or constellation of biomarkers can provide a well-established diagnosis of FTD.

## Conclusions

In summary, our findings indicate that NDEV miRNA profiles are distinct from those derived from plasma circulating miRNAs, suggesting that they could represent an additional resource to identify new biomarkers useful for differential diagnosis between AD and FTD.

## Supplementary Information

Below is the link to the electronic supplementary material.

Supplementary file1 (DOCX 386 KB)

## Data Availability

No datasets were generated or analysed during the current study.

## References

[1] Braak H, Braak E (1997) Frequency of stages of Alzheimer-related lesions in different age categories. Neurobiol Aging 18:351–357. 10.1016/s0197-4580(97)00056-0 9330961 10.1016/s0197-4580(97)00056-0

[2] Cardarelli R, Kertesz A, Knebl JA (2010) Frontotemporal dementia: a review for primary care physicians. Am Fam Physician 82:1372–1377 21121521

[3] Seltman RE, Matthews BR (2012) Frontotemporal lobar degeneration: epidemiology, pathology, diagnosis, and management. CNS Drugs 26:841–870. 10.2165/11640070-000000000-00000 22950490 10.2165/11640070-000000000-00000

[4] Tartaglia MC, Mackenzie IRA (2023) Recent advances in frontotemporal dementia. Can J Neurol Sc 50:485–494. 10.1017/cjn.2022.69 35634749 10.1017/cjn.2022.69

[5] Rabinovici GD, Miller BL (2010) Frontotemporal lobar degeneration: epidemiology, pathophysiology, diagnosis and management. CNS Drugs 24:375–398. 10.2165/11533100-000000000-00000 20369906 10.2165/11533100-000000000-00000 PMC2916644

[6] Yuen SC, Liang X, Zhu H, Jia Y, Leung S-W (2021) Prediction of differentially expressed microRNAs in blood as potential biomarkers for Alzheimer’s disease by meta-analysis and adaptive boosting ensemble learning. Alzheimers Res Ther 13:126. 10.1186/s13195-021-00862-z 34243793 10.1186/s13195-021-00862-z PMC8272278

[7] Piscopo P, Manzini V, Rivabene R, Crestini A, La Pera L, Pizzi E, Veroni C, Talarico G, Peconi M, Castellano AE, D’Alessio C, Bruno G, Corbo M, Vanacore N, Lacorte E (2022) A plasma circular RNA profile differentiates subjects with Alzheimer’s disease and mild cognitive impairment from healthy controls. Int J Mol Sci 23:13232. 10.3390/ijms232113232 36362022 10.3390/ijms232113232 PMC9658433

[8] Grasso M, Piscopo P, Confaloni A, Denti MA (2014) Circulating miRNAs as biomarkers for neurodegenerative disorders. Molecules 19:6891–6910. 10.3390/molecules19056891 24858274 10.3390/molecules19056891 PMC6271879

[9] Turchinovich A, Samatov TR, Tonevitsky AG, Burwinkel B (2013) Circulating miRNAs: cell-cell communication function? Front Genet 4:119. 10.3389/fgene.2013.00119 23825476 10.3389/fgene.2013.00119 PMC3695387

[10] Arora T, Prashar V, Singh R, Barwal TS, Changotra H, Sharma A, Parkash J (2022) Dysregulated miRNAs in progression and pathogenesis of Alzheimer’s disease. Mol Neurobiol 59:6107–6124. 10.1007/s12035-022-02950-z 35867206 10.1007/s12035-022-02950-z

[11] Chu AJ, Williams JM (2022) Astrocytic MicroRNA in ageing, inflammation, and neurodegenerative disease. Front Physiol 12:826697. 10.3389/fphys.2021.826697 35222067 10.3389/fphys.2021.826697 PMC8867065

[12] Tan L, Yu J-T, Hu N, Tan L (2013) Non-coding RNAs in Alzheimer’s disease. Mol Neurobiol 47:382–393. 10.1007/s12035-012-8359-5 23054683 10.1007/s12035-012-8359-5

[13] Piscopo P, Albani D, Castellano AE, Forloni G, Confaloni A (2016) Frontotemporal lobar degeneration and microRNAs. Front Aging Neurosci 8:17. 10.3389/fnagi.2016.00017 26903860 10.3389/fnagi.2016.00017 PMC4746266

[14] Fernández-Messina L, Gutiérrez-Vázquez C, Rivas-García E, Sánchez-Madrid F, de la Fuente H (2015) Immunomodulatory role of microRNAs transferred by extracellular vesicles. Biol Cell 107:61–77. 10.1111/boc.201400081 25564937 10.1111/boc.201400081 PMC5010100

[15] Van Deun J, Mestdagh P, Agostinis P, Akay O, Anand S, Anckaert J, Martinez ZA, Baetens T, Beghein E, Bertier L, Berx G, Boere J, Boukouris S, Bremer M, Buschmann D, Byrd JB, Casert C, Cheng L, Cmoch A, Daveloose D, Smedt E, Demirsoy S, Depoorter V, Dhondt B, Driedonks TAP, Dudek A, Elsharawy A, Floris I, Foers AD, Gärtner K, Garg AD, Geeurickx E, Gettemans J, Ghazavi F, Giebel B, Kormelink TG, Hancock G, Helsmoorte IH, Hill AF, Hyenne V, Kalra H, Kim D, Kowal J, Kraemer S, Leidinger P, Leonelli C, Liang Y, Lippens L, Liu S, Lo Cicero A, Martin S, Mathivanan S, Mathiyalagan P, Matusek T, Milani G, Monguió-Tortajada M, Mus LM, Muth DC, Németh A, Nolte-’t Hoen ENM, O’Driscoll L, Palmulli R, Pfaffl MW, Primdal-Bengtson B, Romano RQ, Sahoo S, Sampaio N, Samuel M, Scicluna B, Soen B, Steels A, Swinnen JV, Takatalo M, Thaminy S, Théry C, Tulkens J, van AudenhoveI I, van der Grein S, Van Goethem A, van Herwijnen MJ, van Niel G, van Roy N, van Vliet AR, Vandamme N, Vanhauwaert S, Vergauwen G, Verweij F, Wallaert A, Wauben M, Witwer KW, Zonneveld MI, de Wever O, Vandesompele J, Hendrix A (2017) EV-TRACK: transparent reporting and centralizing knowledge in extracellular vesicle research. Nat Methods 14:228–232. 10.1038/nmeth.4185 28245209 10.1038/nmeth.4185

[16] Fauré J, Lachenal G, Court M, Hirrlinger J, Chatellard-Causse C, Blot B, Grange J, Schoehn G, Goldberg Y, Boyer V, Kirchhoff F, Raposo G, Garin J, Sadoul R (2006) Exosomes are released by cultured cortical neurons. Mol Cell Neurosci 31:642–648. 10.1016/j.mcn.2005.12.003 16446100 10.1016/j.mcn.2005.12.003

[17] Zhang L, Zhang S, Yao J, Lowery FJ, Zhang Q, Huang WC, Li P, Li M, Wang X, Zhang C, Wang H, Ellis K, Cheerathodi M, McCarty JH, Palmieri D, Saunus J, Lakhani S, Huang S, Sahin AA, Aldape KD, Steeg PS, Yu D (2015) Microenvironment-induced PTEN loss by exosomal microRNA primes brain metastasis outgrowth. Nature 527:100–104. 10.1038/nature15376 26479035 10.1038/nature15376 PMC4819404

[18] Pinto S, Cunha C, Barbosa M, Vaz VR, Brites D (2017) Exosomes from NSC-34 cells transfected with hSOD1-G93A are enriched in miR-124 and drive alterations in microglia phenotype. Front Neurosci 11:273. 10.3389/fnins.2017.00273 28567000 10.3389/fnins.2017.00273 PMC5434170

[19] Doyle LM, Wang MZ (2019) Overview of extracellular vesicles, their origin, composition, purpose, and methods for exosome isolation and analysis. Cells 8:727. 10.3390/cells8070727 31311206 10.3390/cells8070727 PMC6678302

[20] Huang X, Yuan T, Tschannen M, Sun Z, Jacob JH, Du M, Liang M, Dittmar RL, Liu Y, Liang M, Kohli M, Thibodeau SN, Boardman L, Wang L (2013) Characterization of human plasma-derived exosomal RNAs by deep sequencing. BMC Genomics 14:319. 10.1186/1471-2164-14-319 23663360 10.1186/1471-2164-14-319 PMC3653748

[21] Gurunathan S, Kang M-H, Jeyaraj M, Qasim M, Kim J-H (2019) Review of the isolation, characterization, biological function, and multifarious therapeutic approaches of exosomes. Cells 8:307. 10.3390/cells8040307 30987213 10.3390/cells8040307 PMC6523673

[22] Cheng L, Hill AF (2022) Therapeutically harnessing extracellular vesicles. Nat Rev Drug Discov 21:379–399. 10.1038/s41573-022-00410-w 35236964 10.1038/s41573-022-00410-w

[23] Frühbeis C, Fröhlich D, Kuo WP, Krämer-Albers E-M (2013) Extracellular vesicles as mediators of neuron-glia communication. Front Cell Neuroscim 7:182. 10.3389/fncel.2013.00182 10.3389/fncel.2013.00182 PMC3812991 24194697

[24] Rajendran L, Bali J, Barr MM, Court FA, Krämer-Albers E-M, Picou F, Raposo G, van der Vos KE, van Niel G, Wang J, Breakefield XO (2014) Emerging roles of extracellular vesicles in the nervous system. J Neurosci 34:15482–15489. 10.1523/JNEUROSCI.3258-14.2014 25392515 10.1523/JNEUROSCI.3258-14.2014 PMC4228143

[25] Janas AM, Sapoń K, Janas T, Stowell MHB, Janas T (2016) Exosomes and other extracellular vesicles in neural cells and neurodegenerative diseases. Biochim Biophys Acta 1858:1139–1151. 10.1016/j.bbamem.2016.02.011 26874206 10.1016/j.bbamem.2016.02.011

[26] György B, Szabó TG, Pásztói M, Pál Z, Misják P, Aradi B, László V, Pállinger E, Pap E, Kittel A, Nagy G, Falus A, Buzás EI (2011) Membrane vesicles, current state-of-the-art: emerging role of extracellular vesicles. Cell Mol Life Sci 68:2667–2688. 10.1007/s00018-011-0689-3 21560073 10.1007/s00018-011-0689-3 PMC3142546

[27] Ciregia F, Urbani A, Palmisano G (2017) Extracellular vesicles in brain tumors and neurodegenerative diseases. Front Mol Neurosci 10:276. 10.3389/fnmol.2017.00276 28912682 10.3389/fnmol.2017.00276 PMC5583211

[28] Mustapic M, Eitan E, Werner JK Jr, Berkowitz ST, Lazaropoulos MP, Tran J, Goetzl EL, Kapogiannis D (2017) Plasma extracellular vesicles enriched for neuronal origin: a potential window into brain pathologic processes. Front Neurosci 11:278. 10.3389/fnins.2017.00278 28588440 10.3389/fnins.2017.00278 PMC5439289

[29] Goetzl EJ, Abner EL, Jicha GA, Kapogiannis D, Schwartz JB (2018) Declining levels of functionally specialized synaptic proteins in plasma neuronal exosomes with progression of Alzheimer’s disease. FASEB J 32:888–893. 10.1096/fj.201700731R 29025866 10.1096/fj.201700731R PMC5888398

[30] Jiménez-Avalos JA, Fernández-Macías JC, González-Palomo AK (2021) Circulating exosomal MicroRNAs: new non-invasive biomarkers of non-communicable disease. Mol Biol Rep 48:961–967. 10.1007/s11033-020-06050-w 33313972 10.1007/s11033-020-06050-w

[31] Yang Q, Zhao Q, Yin Y (2019) miR-133b is a potential diagnostic biomarker for Alzheimer’s disease and has a neuroprotective role. Exp Ther Med 18:2711–2718. 10.3892/etm.2019.7855 31572518 10.3892/etm.2019.7855 PMC6755445

[32] Lugli G, Cohen AM, Bennett DA, Shah R, Fields CJ, Hernandez AG, Smalheiser NR (2015) Plasma exosomal miRNAs in persons with and without Alzheimer disease: altered expression and prospects for biomarkers. PLoS ONE 10:e0139233. 10.1371/journal.pone.0139233 26426747 10.1371/journal.pone.0139233 PMC4591334

[33] Cheng L, Vella LJ, Barnham KJ, McLean C, Masters CL, Hill AF (2020) Small RNA fingerprinting of Alzheimer’s disease frontal cortex extracellular vesicles and their comparison with peripheral extracellular vesicles. J Extracell Vesicles 9:1766822. 10.1080/20013078.2020.1766822 32922692 10.1080/20013078.2020.1766822 PMC7448944

[34] Piscopo P, Grasso M, Manzini V, Zeni A, Castelluzzo M, Fontana F, Talarico G, Castellano AE, Rivabene R, Crestini A, Bruno G, Ricci L, Denti MA (2023) Identification of miRNAs regulating MAPT expression and their analysis in plasma of patients with dementia. Front Mol Neurosci 16:1127163. 10.3389/fnmol.2023.1127163 37324585 10.3389/fnmol.2023.1127163 PMC10266489

[35] Wang Y, Mandelkow E (2016) Tau in physiology and pathology. Nat Rev Neurosci 17:5–21. 10.1038/nrn.2015.1 26631930 10.1038/nrn.2015.1

[36] Pîrşcoveanu DFV, Pirici I, Tudorică V, Bălşeanu TA, Albu VC, Bondari S, Bumbea AM, Pîrşcoveanu M (2017) Tau protein in neurodegenerative diseases - a review. Rom J Morphol Embryol 58:11411150 29556602

[37] Neary D, Snowden JS, Gustafson L, Passant U, Stuss D, Black S, Freedman M, Kertesz A, Robert PH, Albert M, Boone K, Miller BL, Cummings J, Benson DF (1998) Frontotemporal lobar degeneration: a consensus on clinical diagnostic criteria. Neurology 51:1546–1554. 10.1212/wnl51.6.1546 9855500 10.1212/wnl.51.6.1546

[38] Rascovsky K, Hodges JR, Knopman D, Mendez MF, Kramer JH, Neuhaus J, van Swieten JC, Seelaar H, Dopper EG, Onyike CU, Hillis AE, Josephs KA, Boeve BF, Kertesz A, Seeley WW, Rankin KP, Johnson JK, Gorno-Tempini ML, Rose H, Prioleau-Latham CE, Lee A, Kipps CM, Lillo P, Piguet O, Rohrer JD, Rossor MN, Warren JD, Fox NC, Galasko D, Salmon DP, Black SE, Mesulam M, Weintraub S, Dickerson BC, Diehl-Schmid J, Pasquier F, Deramecourt V, Lebert F, Pijnenburg Y, Chow TW, Manes GJ, Cappa SF, Freedman M, Grossman M, Miller BL (2011) Sensitivity of revised diagnostic criteria for the behavioural variant of frontotemporal dementia. Brain 134:2456–2477. 10.1093/brain/awr179 21810890 10.1093/brain/awr179 PMC3170532

[39] McKhann G, Drachman D, Folstein M, Katzman R, Price D, Stadlan EM (1984) Clinical diagnosis of Alzheimer’s disease: report of the NINCDS-ADRDA Work Group under the auspices of Department of Health and Human Services Task Force on Alzheimer’s Disease. Neurology 34:939–944. 10.1212/wnl34.7.939 6610841 10.1212/wnl.34.7.939

[40] Grasso M, Piscopo P, Talarico G, Ricci L, Crestini A, Tosto G, Gasparini M, Bruno G, Denti MA, Confaloni A (2019) Plasma microRNA profiling distinguishes patients with frontotemporal dementia from healthy subjects. Neurobiol Aging 84:240.e1-240.e12.10.1016/j.neurobiolaging.2019.01.024 30826067 10.1016/j.neurobiolaging.2019.01.024

[41] Piscopo P, Grasso M, Puopolo M, D’Acunto E, Talarico G, Crestini A, Gasparini M, Campopiano R, Gambardella S, Castellano AE, Bruno G, Denti MA, Confaloni A (2018) Circulating miR-127-3p as a potential biomarker for differential diagnosis in frontotemporal dementia. J Alzheimers Dis 65:455–464. 10.3233/JAD-180364 30056425 10.3233/JAD-180364

[42] Cappelletti P, Filareti M, Masuelli L, Bei R, Hassanzadeh K, Corbo M, Feligioni M (2022) Syntaxin-1a and SNAP-25 expression level is increased in the blood samples of ischemic stroke patients. Sci Rep 12:14483. 10.1038/s41598-022-18719-2 36008522 10.1038/s41598-022-18719-2 PMC9411545

[43] Piscopo P, Grasso M, Fontana F, Crestini A, Puopolo M, Del Vescovo V, Venerosi A, Calamandrei G, Vencken SF, Greene CM, Confaloni A, Denti MA (2016) Reduced miR-659-3p levels correlate with progranulin Increase in hypoxic conditions: implications for frontotemporal dementia. Front Mol Neurosci 9:31. 10.3389/fnmol.2016.00031 27199656 10.3389/fnmol.2016.00031 PMC4853935

[44] Andersen CL, Jensen JL, Ørntoft TF (2004) Normalization of real-time quantitative reverse transcription-PCR data: a model-based variance estimation approach to identify genes suited for normalization, applied to bladder and colon cancer data sets. Cancer Res. 64:5245-e5250.10.1158/0008-5472.CAN-04-0496 15289330 10.1158/0008-5472.CAN-04-0496

[45] Vandesompele J, De Preter K, Pattyn F, Poppe B, Van Roy N, De Paepe A, Speleman F. (2002) Accurate normalization of real-time quantitative RT-PCR data by geometric aver aging of multiple internal control genes. Genome Biol 3, RESEARCH0034. 10.1186/gb-2002-3-7-research0034 10.1186/gb-2002-3-7-research0034 PMC126239 12184808

[46] Frühbeis C, Kuo-Elsner WP, Müller C, Barth K, Peris L, Tenzer S, Möbius W, Werner HB, Nave KA, Fröhlich D, Krämer-Albers EM (2022) Oligodendrocytes support axonal transport and maintenance via exosome secretion. PLoS Biol 18(12):e3000621. 10.1371/journal.pbio.3000621 10.1371/journal.pbio.3000621 PMC7787684 33351792

[47] Tallon C, Picciolini S, Yoo SW, Thomas AG, Pal A, Alt J, Carlomagno C, Gualerzi A, Rais R, Haughey NJ, Bedoni M, Slusher BS (2021) Inhibition of neutral sphingomyelinase 2 reduces extracellular vesicle release from neurons, oligodendrocytes, and activated microglial cells following acute brain injury. Biochem Pharmacol. 10.1016/j.bcp.2021.114796 34678224 10.1016/j.bcp.2021.114796 PMC8919377

[48] Verheyen A, Diels A, Reumers J, Van Hoorde K, Van den Wyngaert I, van Outryve DC, De Bondt A, Kuijlaars J, De Muynck L, De Hoogt R, Bretteville A, Jaensch S, Buist A, Cabrera-Socorro A, Wray S, Ebneth A, Roevens P, Royaux I, Peeters PJ (2018) Genetically engineered iPSC-derived FTDP-17 MAPT neurons display mutation-specific neurodegenerative and neurodevelopmental phenotypes. Stem Cell Reports 11:363–379. 10.1016/j.stemcr.2018.06.022 30057263 10.1016/j.stemcr.2018.06.022 PMC6093179

[49] Kopach O, Esteras N, Wray S, Rusakov DA, Abramov AY (2018) Maturation and phenotype of pathophysiological neuronal excitability of human cells in tau-related dementia. J Cell Sci 133:jcs241687. 10.1242/jcs.241687 10.1242/jcs.241687 PMC7272359 32299835

[50] Kopach O, Esteras N, Wray S, Abramov AY, Rusakov DA (2021) Genetically engineered MAPT 10+16 mutation causes pathophysiological excitability of human iPSC-derived neurons related to 4R tau-induced dementia. Cell Death Dis 12:716. 10.1038/s41419-021-04007-w 34274950 10.1038/s41419-021-04007-w PMC8286258

[51] Zeni A, Grasso M, Denti MA (2022) Identification of miRNAs bound to an RNA of interest by MicroRNA capture affinity technology (miR-CATCH). Meth Mol Biol 2404:mv207-218.10.1007/978-1-0716-1851-6_11 10.1007/978-1-0716-1851-6_11 34694611

[52] Li Y, He X, Li Q, Lai H, Zhan H, Hu Z, Li Y, Huang S (2020) EV-origin: enumerating the tissue-cellular origin of circulating extracellular vesicles using exLR profile. Comput Struct Biotechnol J 18:2851–2859. 10.1016/j.csbj.2020.10.002 33133426 10.1016/j.csbj.2020.10.002 PMC7588739

[53] Verheijen J, Sleegers K (2018) Understanding Alzheimer disease at the interface between genetics and transcriptomics. Trends Genet 34:434–447. 10.1016/j.tig.2018.02.007 29573818 10.1016/j.tig.2018.02.007

[54] Alberro A, Iparraguirre L, Fernandes A, Otaegui D (2021) Extracellular vesicles in blood: sources, effects, and applications. Int J Mol Sci 22:8163. 10.3390/ijms22158163 34360924 10.3390/ijms22158163 PMC8347110

[55] D’Anca M, Fenoglio C, Serpente M, Arosio B, Cesari M, Scarpini EA, Galimberti D (2019) Exosome determinants of physiological aging and age-related neurodegenerative diseases. Front Aging Neurosci 11:232. 10.3389/fnagi.2019.00232 31555123 10.3389/fnagi.2019.00232 PMC6722391

[56] Lim WQ, Michelle Luk KH, Lee KY, Nurul N, Loh SJ, Yeow ZX, Wong QX, Daniel Looi QH, Chong PP, How CW, Hamzah S, Foo JB (2023) Small extracellular vesicles’ miRNAs: biomarkers and therapeutics for neurodegenerative diseases. Pharmaceutics 15:1216. 10.3390/pharmaceutics15041216 37111701 10.3390/pharmaceutics15041216 PMC10143523

[57] Vincent B (2022) Plasma extracellular vesicles from the periphery as spreading vectors of Alzheimer’s disease pathogenesis? EBioMedicine 78:103961. 10.1016/j.ebiom.2022.103961 35325782 10.1016/j.ebiom.2022.103961 PMC8938881

[58] Yang TT, Liu CG, Gao SC, Zhang Y, Wang PC (2018) The serum exosome derived MicroRNA-135a, -193b, and -384 were potential Alzheimer’s disease biomarkers. Biomed Environ Sc 31:87–96. 10.3967/bes2018.011 29606187 10.3967/bes2018.011

[59] Wei H, Xu Y, Xu W, Zhou Q, Chen Q, Yang M, Feng F, Liu Y, Zhu X, Yu M, Li Y (2018) Serum exosomal miR-223 serves as a potential diagnostic and prognostic biomarker for dementia. Neuroscience 379:167–176. 10.1016/j.neuroscience.2018.03.016 29559383 10.1016/j.neuroscience.2018.03.016

[60] Ryu IS, Kim DH, Ro JY, Park BG, Kim SH, Im JY, Lee JY, Yoon SJ, Kang H, Iwatsubo T, Teunissen CE, Cho HJ, Ryu JH (2023) The microRNA-485-3p concentration in salivary exosome-enriched extracellular vesicles is related to amyloid β deposition in the brain of patients with Alzheimer’s disease. Clin Biochem 118:110603. 10.1016/j.clinbiochem.2023.110603 37355215 10.1016/j.clinbiochem.2023.110603

[61] Visconte C, Fenoglio C, Serpente M, Muti P, Sacconi A, Rigoni M, Arighi A, Borracci V, Arcaro M, Arosio B, Ferri E, Golia MT, Scarpini E, Galimberti D (2023) Altered extracellular vesicle miRNA profile in prodromal Alzheimer’s disease. Int J Mol Sci 24:14749. 10.3390/ijms241914749 37834197 10.3390/ijms241914749 PMC10572781

[62] Schneider R, McKeever P, Kim T, Graff C, van Swieten JC, Karydas A, Boxer A, Rosen H, Miller BL Jr, Laforce R, Galimberti D, Masellis M, Borroni B, Zhang Z, Zinman L, Rohrer D, Tartaglia MC, Robertson J (2018) Genetic FTD Initiative (GENFI). Downregulation of exosomal miR-204-5p and miR-632 as a biomarker for FTD: a GENFI study. J Neurol Neurosurg Psychiatry 89:851–858. 10.1136/jnnp-2017-317492 29434051 10.1136/jnnp-2017-317492 PMC6045452

[63] Pounders J, Hill EJ, Hooper D, Zhang X, Biesiada J, Kuhnell D, Greenland HL, Esfandiari L, Timmerman E, Foster F, Wang C, Walsh KB, Shatz R, Woo D, Medvedovic M, Langevin S, Sawyer RP (2022) MicroRNA expression within neuronal-derived small extracellular vesicles in frontotemporal degeneration. Medicine 101:e30854. 10.1097/MD.0000000000030854 36221381 10.1097/MD.0000000000030854 PMC9542922

[64] Pishbin E, Sadri F, Dehghan A, Kiani MJ, Hashemi N, Zare I, Mousavi P, Rahi A (2023) Recent advances in isolation and detection of exosomal microRNAs related to Alzheimer’s disease. Environ Res 227:115705. 10.1016/j.envres.2023.115705 36958383 10.1016/j.envres.2023.115705

[65] Song S, Lee JU, Jeon MJ, Kim S, Lee CN, Sim SJ (2023) Precise profiling of exosomal biomarkers via programmable curved plasmonic nanoarchitecture-based biosensor for clinical diagnosis of Alzheimer’s disease. Biosens Bioelectron 230:115269. 10.1016/j.bios.2023.115269 37001292 10.1016/j.bios.2023.115269

[66] Oeckl P, Steinacker P, Feneberg E, Otto M (2016) Neurochemical biomarkers in the diagnosis of frontotemporal lobar degeneration: an update. J Neurochem 138(Suppl 1):184–92. 10.1111/jnc.13669 27186717 10.1111/jnc.13669

[67] Liu MN, Lau CI, Lin CP (2019) Precision medicine for frontotemporal dementia. Front Psychiatry 10:75. 10.3389/fpsyt.2019.00075 30846947 10.3389/fpsyt.2019.00075 PMC6393374

[68] Hedl TJ, San Gil R, Cheng F, Rayner SL, Davidson JM, De Luca A, Villalva MD, Ecroyd H, Walker AK, Lee A (2019) Proteomics approaches for biomarker and drug target discovery in ALS and FTD. Front Neurosci 13:548. 10.3389/fnins.2019.00548 31244593 10.3389/fnins.2019.00548 PMC6579929

[69] Del Campo M, Zetterberg H, Gandy S, Onyike CU, Oliveira F, Udeh-Momoh C, Lleó A, Teunissen CE, Pijnenburg Y (2022) New developments of biofluid-based biomarkers for routine diagnosis and disease trajectories in frontotemporal dementia. Alzheimers Dement 18:2292–2307. 10.1002/alz.12643 35235699 10.1002/alz.12643 PMC9790674

