# Supplementary material for: miR-92a-3p and miR-320a are Upregulated in Plasma Neuron-Derived Extracellular Vesicles of Patients with Frontotemporal Dementia
Source: Mol Neurobiol. 2024 Aug 14;62(2):2573–86. doi: 10.1007/s12035-024-04386-z (PMC11772464; doi:10.1007/s12035-024-04386-z)
Supplement: Supplementary file 1 — Supplementary file1 (DOCX 386 KB) [file 12035_2024_4386_MOESM1_ESM.docx]

**Supplementary Material**

**Supplementary Table 1.** **Concentration of RNA isolated from cellular culture.** RNA was purified from wild type (WT) and mutant (mut) neurons cultured for 120 days (d120). Four biological replicates were performed (rep # 1-4). RNA concentration and OD ratios were measured by NanoDrop ND-1000).

| **Samples** | **Concentration (ng/µl)** | **OD 260/280** | **OD 260/230** |
| --- | --- | --- | --- |
| d120 WT rep #1 | 168.0 | 1.83 | 1.55 |
| d120 mut rep #1 | 103.8 | 1.75 | 1.63 |
| d120 WT rep #2 | 253.3 | 1.82 | 1.98 |
| d120 mut rep #2 | 308.5 | 1.87 | 2.05 |
| d120 WT rep #3 | 187.3 | 1.85 | 1.40 |
| d120 mut rep #3 | 176.6 | 1.81 | 1.75 |
| d120 WT rep #4 | 228.1 | 1.83 | 1.13 |
| d120 mut rep #4 | 166.8 | 1.86 | 1.32 |

**Supplementary Table 2. Quantitative PCR primers for the analysis of microRNAs in hiPSCs**

| **Primer name** | **Company** | **Cataloguenumber** | **Assay ID** |
| --- | --- | --- | --- |
| hsa-miR-320a-3p | Thermo Fisher | 4427975 | 002277 |
| hsa-miR-320b | Thermo Fisher | 4427975 | 002844 |
| hsa-miR-92a-3p | Thermo Fisher | 4427975 | 000431 |
| RNU48 | Thermo Fisher | 4427975 | 001006 |

**
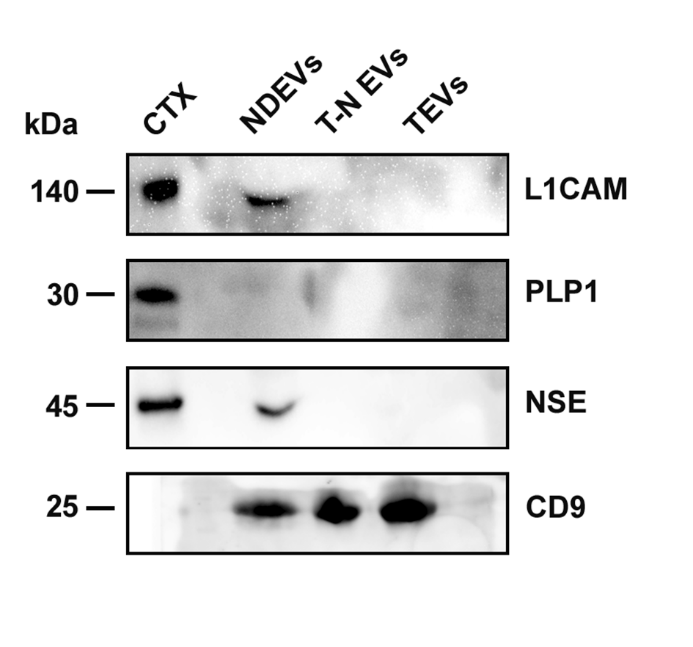
**

**Supplementary Figure 1.** **Quality control of NDEVs purification from plasma of CT subject.** A representative western blot showing the detection of L1CAM, a protein used as neuronal marker, PLP1, a protein used as oligodentroglial marker, NSE, and CD9 commonly used as an exosome marker. Mouse brain cortex lysate (CTX) has been used as positive control. The experiment has been run in triplicate.

**Supplementary Table 3. Number of EVs extracted in NDEVs and TEVs.** Each value is shown as % ± SE of EVs number (expressed as % to the CT group). The p-values corresponding to each comparison were obtained by T-test.

|  |  | **NSE**  **(% vs CT ± SE)** | **P_value vs AD** | **P_value vs FTD** | **CD9**  **(% vs CT ± SE)** | **P_value vs AD** | **P_value vs FTD** |
| --- | --- | --- | --- | --- | --- | --- | --- |
| **NDEVs** | **CT** | 100%±29.3 | 0.240 | 0.924 | 98.6% ± 25.8 | 0.577 | 0.986 |
|  | **AD** | 117% ± 28.0 |  | 0.427 | 107% ± 19.3 |  | 0.477 |
|  | **FTD** | 104% ± 24.5 |  |  | 97.3% ± 17.6 |  |  |
| **TEVs** | **CT** |  |  |  | 98.7% ± 22.2 | 0.126 | 0.461 |
|  | **AD** |  |  |  | 83.7% ± 16.3 |  | 0.727 |
|  | **FTD** |  |  |  | 89.6% ± 19.0 |  |  |

**Supplementary Table 4. miRNAs expression levels in NDEVs from plasma samples**. Each value is shown as mean ± Standard error (SE) of 2^-ΔCt.^ The p-values corresponding to each comparison were obtained by T-test.

|  | **miR-92a-3p**  **(Mean±SE)** | **P_value vs CT** | **P_value vs AD** | **miR-320a**  **(Mean±SE)** | **P_value vs CT** | **P_value vs AD** | **miR-320b**  **(Mean±SE)** | **P_value vs CT** | **P_value vs AD** |
| --- | --- | --- | --- | --- | --- | --- | --- | --- | --- |
| **CT** | 0.200±0.03 |  |  | 0.145±0.03 |  |  | 4.503±0.89 |  |  |
| **AD** | 0.198±0.03 | 0.957 |  | 0.228±0.03 | 0.195 |  | 3.570±1.17 | 0.537 |  |
| **FTD** | 0.605±0.17 | 0.026 | 0.020 | 0.578±0.08 | <0.001 | 0.001 | 5.639±1.41 | 0.490 | 0.266 |

**Supplementary Table 5. miRNAs expression levels in TEVs from plasma samples.** Each value is shown as mean ± Standard error (SE) of 2^-ΔCt^. The p-values corresponding to each comparison were obtained by T-test.

|  | **miR-92a-3p**  **(Mean±SE)** | **P_value vs CT** | **P_value vs AD** | **miR-320a**  **(Mean±SE)** | **P_value vs CT** | **P_value vs AD** | **miR-320b**  **(Mean±SE)** | **P_value vs CT** | **P_value vs AD** |
| --- | --- | --- | --- | --- | --- | --- | --- | --- | --- |
| **CT** | 1.474±0.27 |  |  | 1.105±0.11 |  |  | 0.073±0.01 |  |  |
| **AD** | 0.580±0.10 | 0.004 |  | 0.441±0.17 | 0.003 |  | 0.079±0.01 | 0.719 |  |
| **FTD** | 0.629±0.18 | 0.014 | 0.809 | 1.303±0.29 | 0.527 | 0.016 | 0.141±0.03 | 0.031 | 0.065 |

**Supplementary Table 6. miRNAs expression levels from CSF samples.** Each value is shown as mean ± Standard error (SE) of 2^-ΔCt^.

|  | **miR-92a-3p**  **(Mean±SE)** | **P_value vs CT** | **P_value vs AD** | **miR-320a**  **(Mean±SE)** | **P_value vs CT** | **P_value vs AD** | **miR-320b**  **(Mean±SE)** | **P_value vs CT** | **P_value vs AD** |
| --- | --- | --- | --- | --- | --- | --- | --- | --- | --- |
| **CT** | 0.160±0.01 |  |  | 0.160±0.03 |  |  | 4.478±0.38 |  |  |
| **AD** | 0.262±0.10 | 0.365 |  | 0.527±0.20 | 0.108 |  | 5.108 0.34 | 0.112 |  |
| **FTD** | 0.502±0.15 | 0.021 | 0.189 | 0.877±0.29 | 0.013 | 0.320 | 4.205±0.42 | 0.365 | 0.229 |


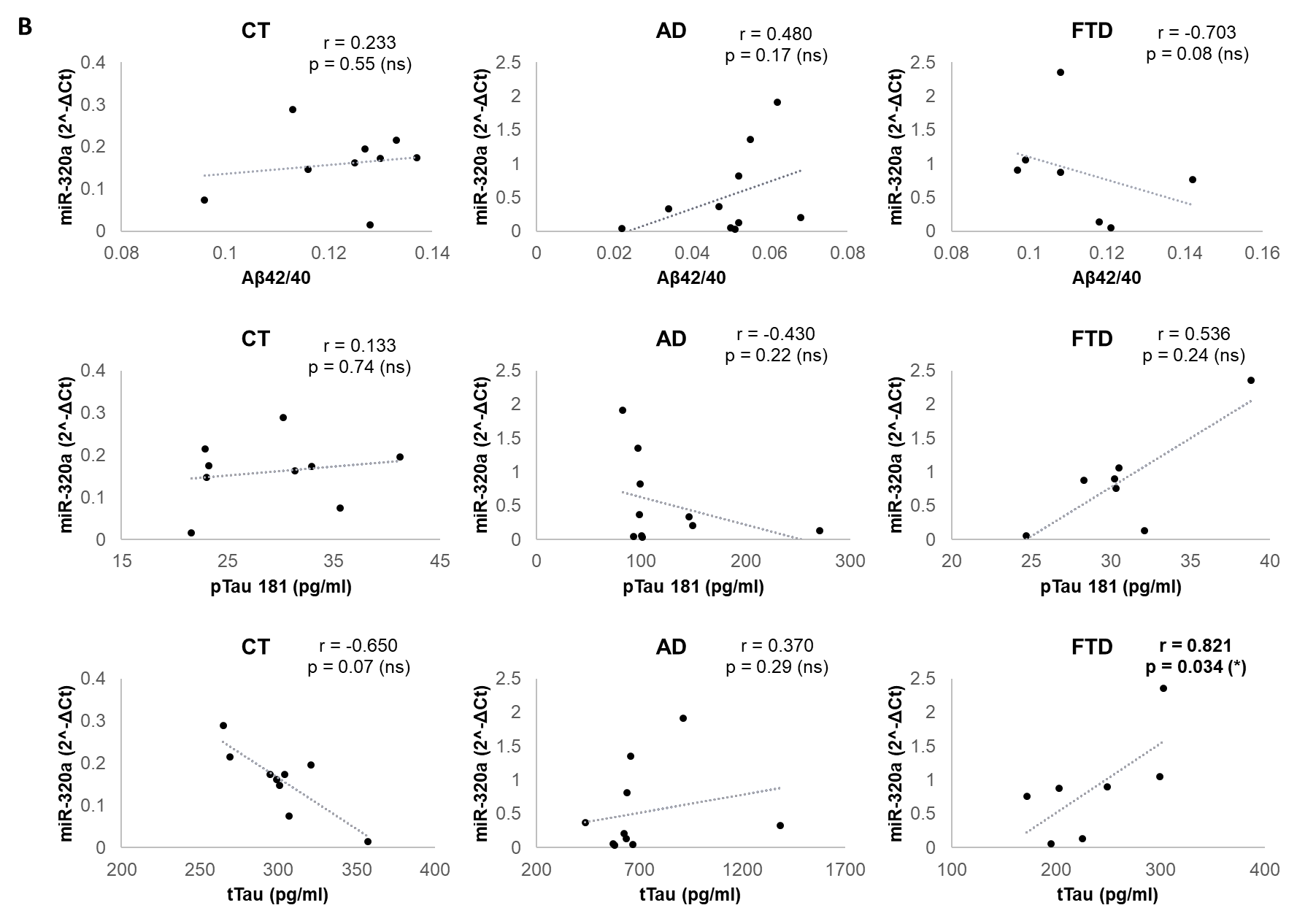

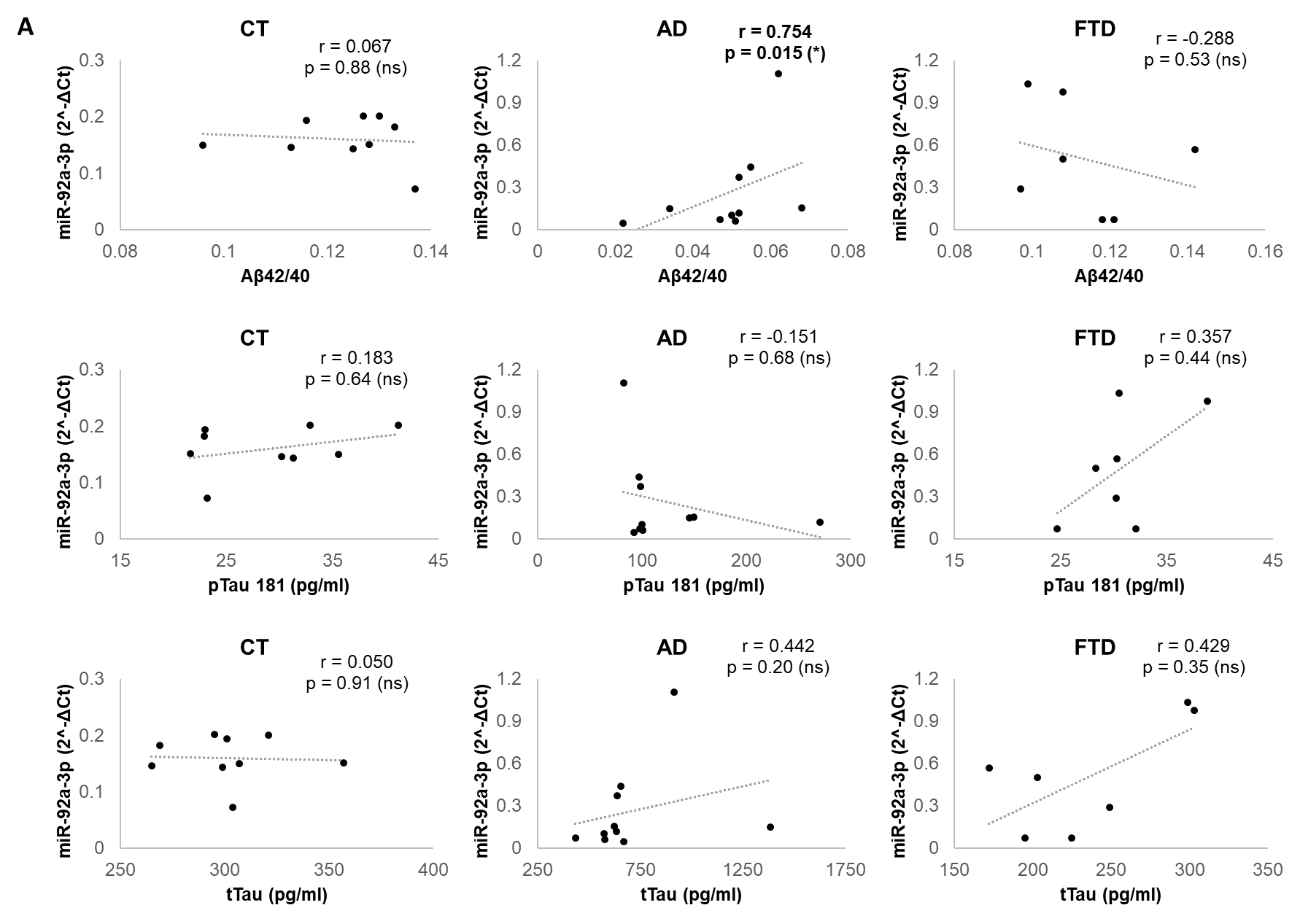


**
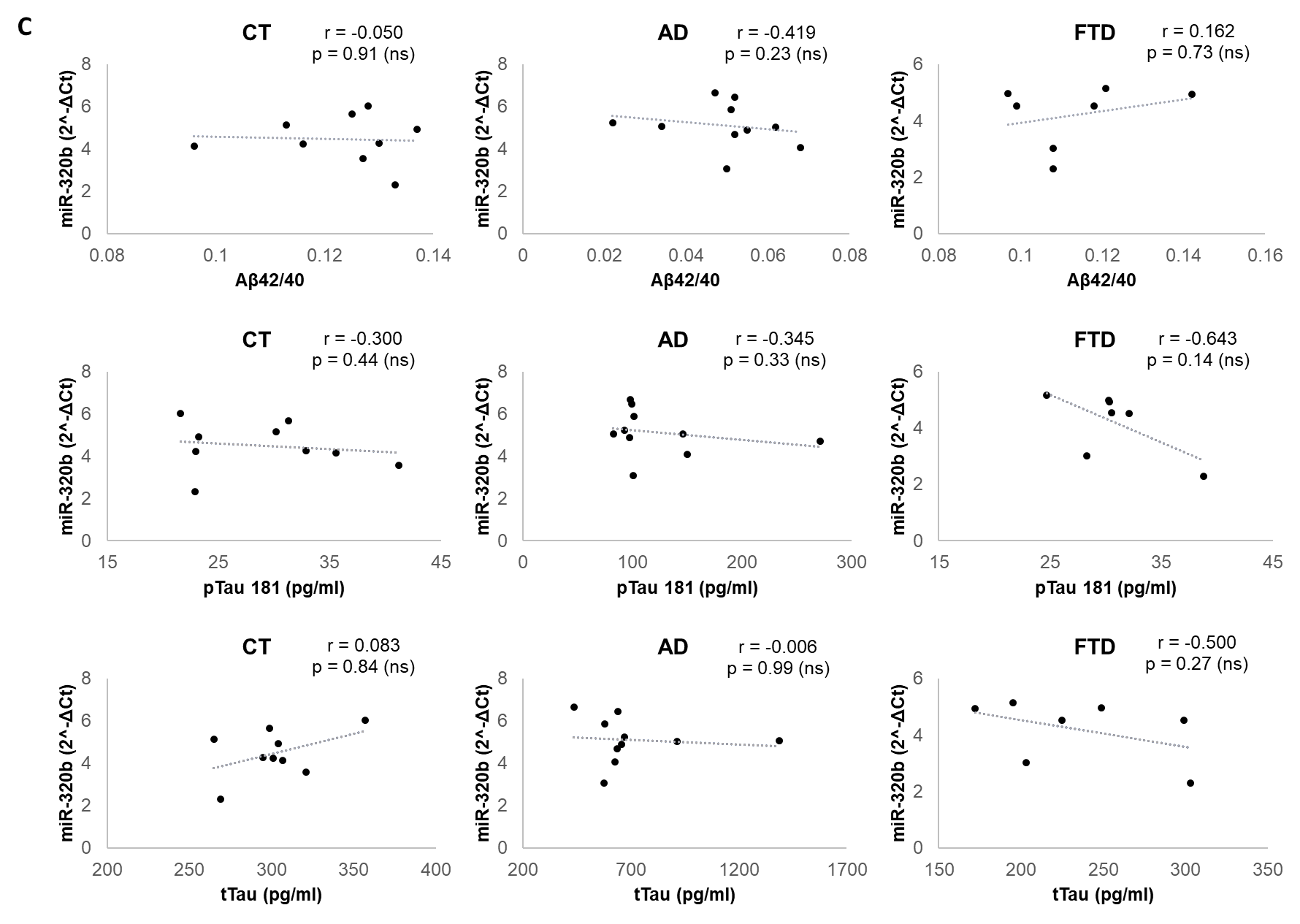
Supplementary Figure 2: Correlations between miRNAs levels and AD protein biomarkers in CSF.** The graphs represent the correlations between the protein fluid biomarkers Aβ42/40 ratio, pTau181 and tTau concentrations (pg/ml) and the miR-92a-3p (A), miR-320a (B) and miR-320b (C) levels in CSF. The population was divided into the three groups: AD, FTD and CT. r = Spearman correlation coefficient. p_value was considered as significant if < 0.05 (*).
